# Influence of yttrium doping on the nonlinear optical limiting properties of cadmium molybdate nanostructures†

**DOI:** 10.1039/d2ra04687c

**Published:** 2022-09-23

**Authors:** B. Binish, K. Mani Rahulan, A. Dhanusha, T. C. Sabari Girisun, Junaid Masud Laskar

**Affiliations:** Nanophotonics Research Laboratory, Department of Physics & Nanotechnology, SRM Institute of Science and Technology Kattankulathur Tamilnadu 603 203 India krahul.au@gmail.com jmlaskar@gmail.com; Nanophotonics Laboratory, Department of Physics, Bharathidasan University Tiruchirappalli 620 024 India

## Abstract

The emerging demand for the production of nonlinear optical materials with high optical limiting performance has an apparent impact in the field of nonlinear optics owing to their wide application in photonic devices. In this regard, transition metal molybdates have received attention owing to their remarkable optical and luminescence characteristics, leading to their extensive use in next generation optoelectronics devices. Herein, we report the nonlinear optical response of yttrium (Y^3+^) doped cadmium molybdate (CdMoO_4_) nanostructures synthesized *via* a co-precipitation technique. The X-ray diffraction and Raman spectroscopy results confirm the formation of CdMoO_4_ nanostructures with a tetragonal structure having the I4_1_/*a* space group. High resolution scanning electron microscopy (HRSEM) of the pristine CdMoO_4_ exposed the cubic flat edged nature of the nanostructures and that doping results in particle size reduction due to lattice contraction. X-ray photo electron spectroscopy confirmed the chemical state of the elements present in Y^3+^doped CdMoO_4_. The optical properties of the samples were studied using UV-Vis Spectroscopy and the bandgap was found to increase upon Y^3+^ doping. The NLO response measured using the open aperture z-scan technique with a Nd: YAG pulsed laser (532 nm, 7 ns, 10 Hz) exhibited a reverse saturable absorption arising from a two photon absorption (2PA) process. An increase in the 2PA coefficient and simultaneous decrease in the onset of the optical limiting threshold clearly suggests the great potential of the yttrium-doped CdMoO_4_ nanoparticles for good optical limiting applications.

## Abstract 2

(a) Polyhedral structure and (b) two photon absorption of CdMoO4 nanostructures.

## Graphical abstract

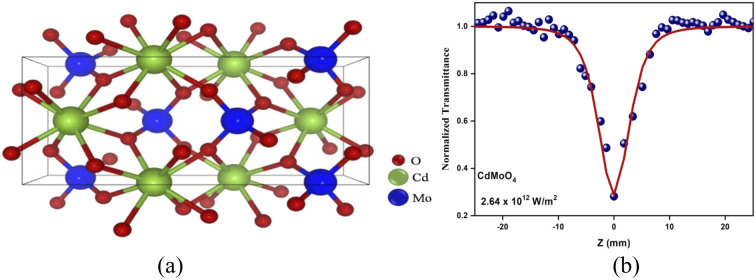

## 1. Introduction

Novel optical materials with ultrafast response times, good nonlinearity and optical limiting have been developed and found a wide range of applications in data storage, frequency conversion, optical switches, electro-optics and optical limiting.^1–4^ Due to the tremendous increase in the use of high intensity pulsed laser, the protection of delicate optical components from laser induced damage has become significant. In this regard, optical limiting action achieved through two photon absorption (2PA), excited state absorption (ESA), free carrier absorption (FCA), nonlinear refraction, thermal defocusing and induced scattering have gained much attention.^5^ These nonlinear optical (NLO) properties arising through photon–photon interactions can induce modulation in the light intensity, which creates opportunities to design laser safety devices and in light filtering/shielding applications.^6^ Hence, the search for novel and effective nonlinear optical limiting materials has covered various organic and inorganic materials.^7^ As organic materials have poor thermal and mechanical stability, the risk of damage is high when exposed to high intensity laser whereas the inorganic materials have attracted attention owing to their distinctive optical and nonlinear properties in addition to their high thermal stability. Hence, inorganic transition metals exhibiting high thermal stability, and optical and nonlinear properties have recently attracted attention.^8^

Among the inorganic materials, transition metal molybdates have attracted significant attention due to their remarkable structural, optical and luminescence properties.^9^ The scheelite type metal molybdates have been used for various applications such as laser materials,^10^ scintillation detectors^11^ and photoluminescent devices^12^ owing to their excellent chemical and physical properties. Studies on metal molybdates, such as CoMoO_4,_ BaMoO_4_ and SrMoO_4,_ exhibiting a high damage threshold and nonlinearity have opened a new channel for developing optical and optoelectronic devices.^13–15^ Of the transition metal molybdates, cadmium molybdate has received special attention owing to its exceptional electronic, chemical and optical properties.^16,17^ In order to tailor the band gap as well as the optical properties, doping has become an effective technique; by the incorporation of a proper dopant, the optical properties can be tuned. Doping of rare earth metals has been found to enhance the optical properties, based on the energy level transition (4f-4f or 5d-4f), of the host material, which has provided potential in the display and lighting fields.^18–20^ Metal molybdates can be synthesised in a number of ways, such as the hydrothermal method,^21^ solid state synthesis,^22^ mechanical synthesis, and the sol gel technique.^23^ Among these, the co-precipitation synthesis technique has been found to be the most effective and cheapest method for the synthesis of metal molybdates. To the best of our knowledge, the optical limiting properties of yttrium (Y^3+^) doped cadmium molybdate (CdMoO_4_) have not yet been reported. Herein, we report the nonlinear absorption and optical limiting properties of yttrium doped cadmium molybdate nanostructures, investigated using a nanosecond Q switched pulsed (7 ns) Nd:YAG laser at 532 nm wavelength with the z-scan method.^24,25^

## 2. Synthesis of yttrium (Y^3+^) doped cadmium molybdate (CdMoO_4_)

The typical chemical synthesis technique was followed for preparing Yttrium (Y^3+^) doped cadmium molybdate (CdMoO_4_). 0.2 M cadmium nitrate tetrahydrate and 0.2 M sodium molybdate dehydrate are placed in separate beakers with 10 ml of distilled water under constant stirring. Different molar concentrations of yttrium (0.1, 0.3, and 0.5%) dissolved in 10 ml of deionized water are added dropwise to the Cd^2+^ solution under stirring for 30 min. The as-prepared Cd^2+^/Y^3+^solution is then added dropwise to the molybdate solution and left to stir for 1 hour followed by 12 hours of aging. The obtained product is centrifuged several times with ethanol and distilled water to remove the byproducts. Next, the precipitate is dried at 60 °C overnight followed by annealing at 550 °C for 3 h. The same procedure was followed for the preparation of pristine cadmium molybdate without any addition of yttrium ions.

## 3. Results and discussion

### 3.1 X-Ray diffraction analysis

**Fig. 1:**
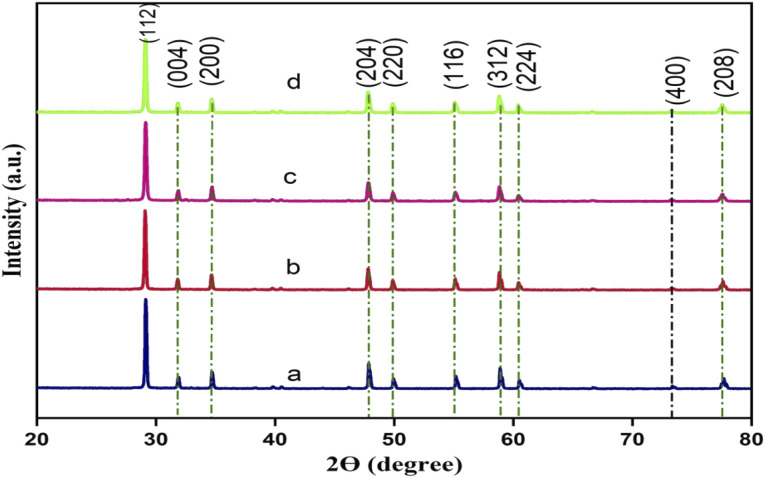
XRD patterns of Y^3+^ doped CdMoO_4_ nanoparticles: (a) pure CdMoO_4_, and (b) 0.1%, (c) 0.3%, and (d) 0.5% Y^3+^-doped CdMoO_4_.

The crystalline phase and structure of the synthesised pristine CdMoO_4_ and yttrium (Y^3+^) doped CdMoO_4_ nanostructures were analysed using powder X-ray diffraction, as shown in Fig. 1. The pristine CdMoO_4_ nanoparticles have scheelite type symmetry with a space group of I4_1_/*a*, in good concurrence with the JCPDS card number 00-007-0209.^26^ All of the XRD patterns show sharp and intense peaks, indicating the strongly crystalline nature of the material. The substitution of Y^3+^ ions into the CdMoO_4_ lattice slightly reduces the diffraction peak intensity, resulting in a reduction of crystallinity. No other impurity peaks corresponding to Y_2_O_3_ or YO_3_ were observed, revealing that Y^3+^ doping has no apparent influence on crystal orientation.^17,27^ The average crystallite size of the prepared samples was calculated using the Scherer formula and found to be around 43.5, 43.3, 36.1 and 33.2 nm for pristine CdMoO_4_, and 0.1%, 0.3% and 0.5% yttrium doped CdMoO_4,_ respectively.

### 3.2 Raman and FTIR spectroscopy

Raman spectroscopy was used for further confirmation of the crystal structure as well as the bonding nature of the yttrium (Y^3+^) doped cadmium molybdate (CdMoO_4_) nanostructures. It is well known that the tetragonal scheelite structure of CdMoO_4_ has S4 symmetry and from group theory, the Γ point Brillouin zone can be expressed by 26 lattice vibrations asΓ = 3A_g_ + 5A_u_ + 5B_g_ + 3B_u_ + 5E_g_+ 5E_u_

**Fig. 2:**
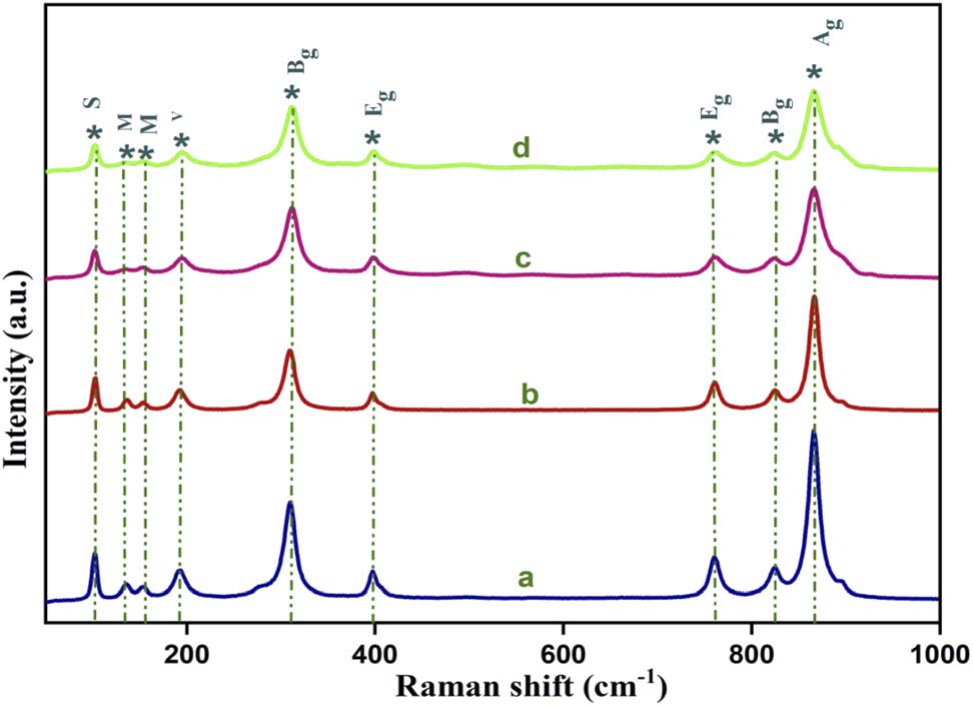
Raman spectra of Y^3+^ doped CdMoO_4_ nanoparticles: (a) pure CdMoO_4_, and (b) 0.1%, (c) 0.3%, and (d) 0.5% Y-doped CdMoO_4_.

Out of these 26 modes, 3A_g_, 5B_g_, and 5E_g_ are Raman active whereas 4A_u_ and 4E_u_ are IR active. 3Bu is known as the silent mode and the remaining 1A_u_ and 1E_u_ are two acoustic modes that are IR and Raman inactive. The Raman active modes are further categorized as seven internal modes, due to internal stretching and bending vibration, and six external modes corresponding to three rotation modes, two transitional and unidentified modes.^28^Fig. 2 shows the Raman spectrum of the yttrium (Y^3+^) doped cadmium molybdate (CdMoO_4_) nanostructures. The sharp and intense peak at 866.8 cm^−1^ can be attributed to the *v*_1_(A_g_) symmetric stretching vibration of Mo–O bonds. The anti-symmetric *v*_3_(B_g_) and *v*_3_(E_g_) vibration of Mo–O bonds is observable at 825.1 and 760.1 cm^−1^. Similarly, the peaks at 396.9 and 309.3 cm^−1^ correspond to the stronger *v*_2_(A_g_) and weaker *v*_4_(B_g_) modes. The peaks observed at 193.3, 152.7, 136.2 and 102.3 cm^−1^ can be attributed to the regular (MoO_4_)^2−^ tetrahedrons. All of the peaks were identified and matches well with the reported values.^29–32^ The decrease in peak intensity of the Raman spectrum on doping confirms the proper substitution of Y^3+^ into the CdMoO_4_ lattice.

**Fig. 3:**
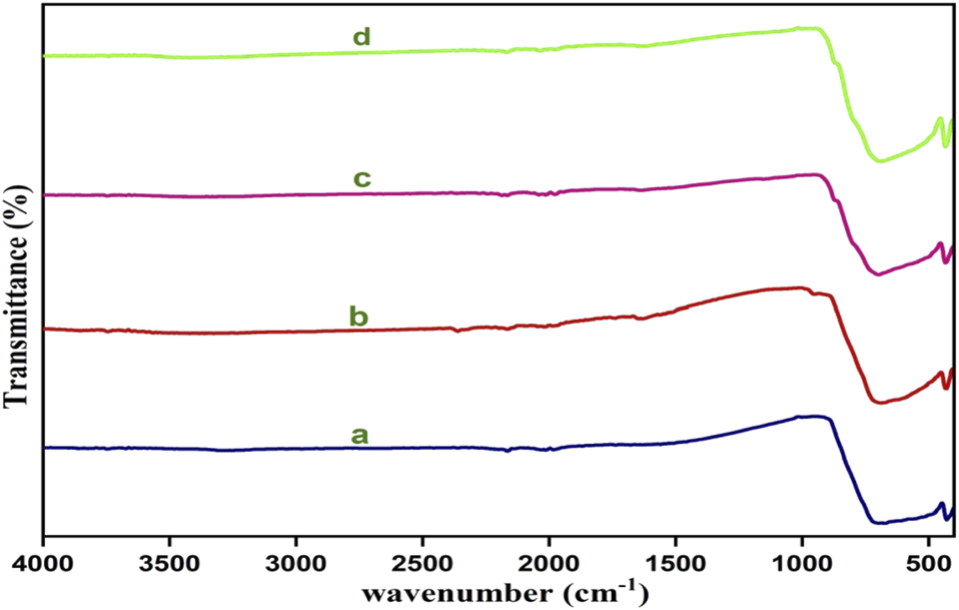
FTIR Spectra of Y^3+^ doped CdMoO_4_ nanoparticles: (a) pure CdMoO4, and (b) 0.1%, (c) 0.3%, and (d) 0.5% Y^3+^-doped CdMoO_4_.

Fig. 3 shows the FTIR spectra of yttrium (Y^3+^) doped cadmium molybdate (CdMoO_4_) for different doping contents of Y^3+^ ions. The spectra show a strong transmittance at 720–892 cm^−1^, owing to the anti-symmetric stretching vibration of [MoO_4_]^2−^ tetrahedrons, and a peak at 430 cm^−1^, which is attributed to asymmetric vibration due to the Mo–O bending mode. These modes are in good accordance with the results reported in previously obtained findings.^33–35^

### 3.3 High resolution scanning electron microscopy (HRSEM)

**Fig. 4:**
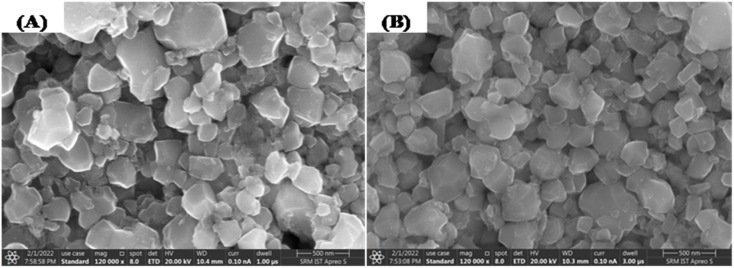
HRSEM images of (A) pure CdMoO_4_ and (B) 0.3% Y^3+^-doped CdMoO_4_ nanoparticles.

The surface morphology and chemical composition of the yttrium doped cadmium molybdate nanoparticles were studied using high resolution scanning electron microscopy (HRSEM). Fig. 4A and B show the surface morphology images of the (Y^3+^) doped CdMoO_4_ nanoparticles showing round edged cubic individual particles with a tendency of agglomeration. On increasing the dopant concentration, the particle size decreases with a similar morphology, which clearly suggests that yttrium ions have an impact on lattice contraction and do not influence crystal structure.

### 3.4 X-Ray photoelectron spectroscopy (XPS)

**Fig. 5:**
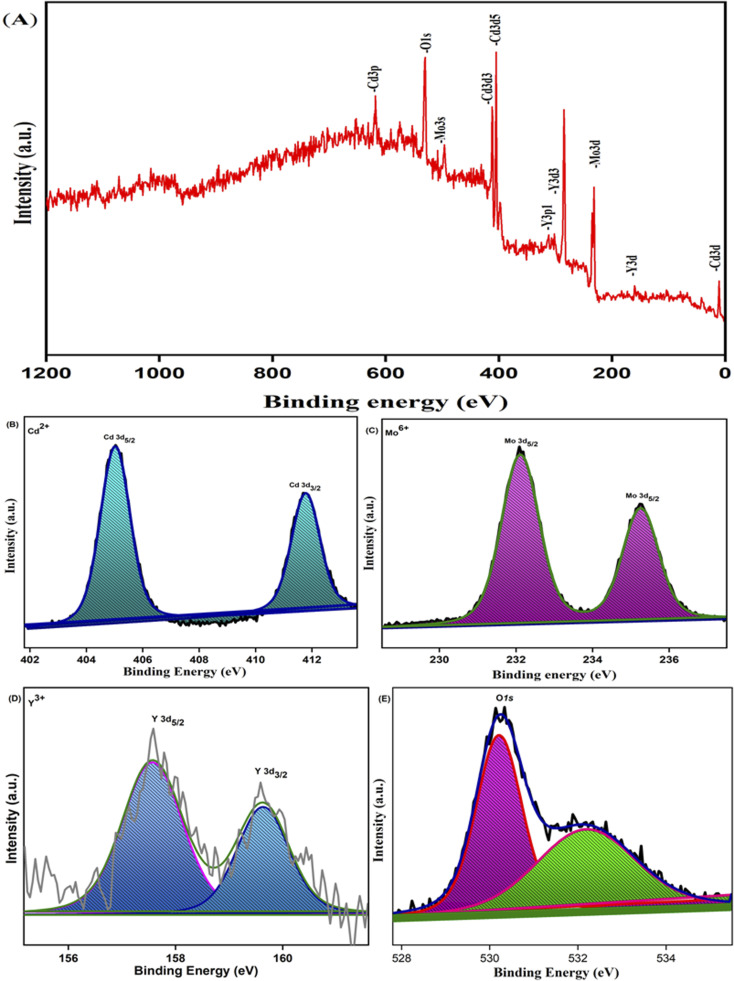
XPS spectra of 0.3% Y^3+^doped CdMoO_4_: (A) survey spectrum, (B) Cd 3d spectrum, (C) Mo 3d spectrum, (D) Y 3d spectrum and (E) O 1s spectrum.

X-Ray photoelectron spectroscopy analysis confirmed the surface composition and oxidation state of the 0.3% Y-doped CdMoO_4_ nanostructures. The core level binding energy and survey spectrum of the sample is depicted in Fig. 5(A). The survey spectrum shows that there are no impurity peaks except the C 1s peak at a binding energy of around 284.5 eV that is due to the hydrocarbon present within the XPS instrument (Fig. 5A). The core level spectrum of the Cd 3d state of cadmium (Fig. 5B) shows two prominent peaks at 405 and 411.7 eV with a splitting width of 6.7 eV attributed to Cd 3d_5/2_ and Cd 3d_3/2_ with the 2+ oxidation state of the material (Cd^2+^). The core level spectrum of Mo 3d was deconvoluted into two peaks at binding energies of 232.0 eV corresponding to Mo 3d_5/2_ and at 235.2 eV corresponding to Mo 3d_3/2_ with a splitting width of 3.2 eV, attributed to the Mo^6+^ oxidation state, as depicted in Fig. 5C, which matches well with existing literature.^36,37^ The XPS spectrum of yttrium was observed over a range of 155 to 161 eV, as shown in Fig. 5D; the deconvoluted peak at 157.6 eV represents Y 3d_5/2_ and the peak at 159.6 eV represents Y 3d_3/2_ with a splitting width of 2 eV corresponding to the two spin orbit peak of the Y^3+^ state.^38,39^ The peaks at 530.2 eV and 532.1 eV indicate the O 1s (oxygen) state, as shown in Fig. 5E. The peak at 530.2 eV is considered to have originated from O^2−^ anions in the stoichiometric CdMoO_4_ structure and oxygen vacancies on the surface of the sample, while the peak at 532.1 eV corresponds to water molecules or absorbed oxygen species, which also indicates the presence of Cd–O bonds in the CdMoO_4_ structure.

### 3.5 UV-Vis absorption spectroscopy

**Fig. 6:**
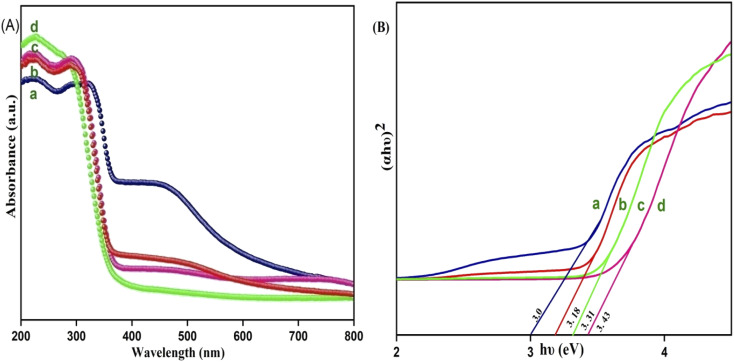
(A) UV DRS Spectrum and (B) optical band gap of Y^3+^ doped CdMoO_4_ nanoparticles: (a) pure CdMoO_4_, (b) 0.1%, (c) 0.3% and (d) 0.5% Y^3+^–CdMoO_4_.

The optical properties of the Y^3+^ doped CdMoO_4_ nanostructures were investigated using UV-Vis absorption spectroscopy and the recorded spectra are shown in Fig. 6A. All of the samples with different concentrations of Y in CdMoO_4_ exhibited exemplary absorption in the UV region, showing maximum absorption in the 200–300 nm region. In CdMoO_4_, the valence band is composed of 2p orbitals, which are occupied, and the conduction band consists of empty Mo 4d orbitals. The broad absorption in the UV region can be directly explained by the charge carrier mechanism from oxygen electrons in the 2p orbitals to (MoO_4_)^2+^ ions inside the central molybdenum atom.^40^ It was observed that as the yttrium doping concentration increases, the visible (400–800 nm) absorption of pure CdMoO_4_ decreases and it becomes negligible at higher concentrations of yttrium. From the absorption data, the band gap (E_g_) can be calculated using Tauc plot by extrapolating a linear plot between the photon energy (*hυ*) along the abscissas and (*αhυ*)^2^ along the ordinates, as shown in Fig. 6B. The band gap (E_g_) of CdMoO_4_ was found to be 3.27 eV and it increases to 3.36, 3.44, and 3.5 eV for 0.1, 0.3 and 0.5% Y–CdMoO_4_, which agrees with the literature. Quantum confinement theory suggests that the surface potential barrier spatially confines the electrons inside the conduction band and holes within the valence band. So, the lowest optical energy transition from the valence band to the conduction band increases in energy owing to electron hole confinement, resulting in the increase in band gap.^24,27^

### 3.6 Z-scan analysis

**Fig. 7:**
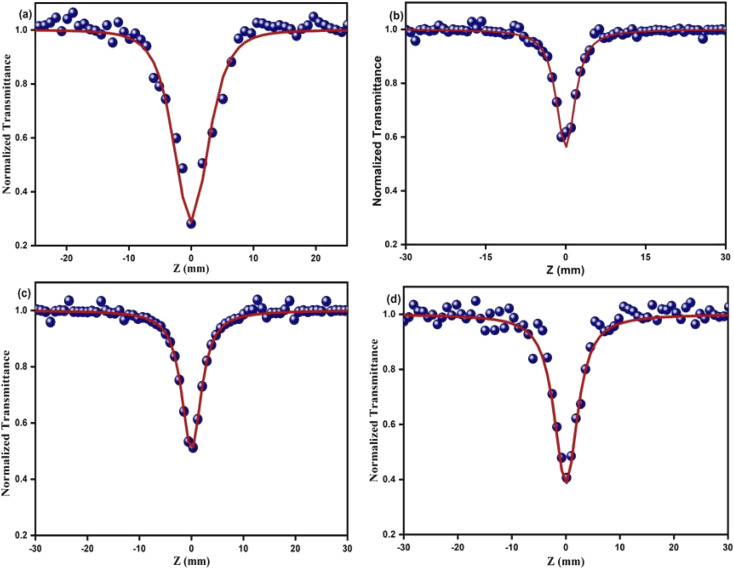
Open aperture Z-scan traces of Y^3+^ doped CdMoO_4_ nanostructures: (a) pure CdMoO_4_, (b) 0.1%, (c) 0.3% and (d) 0.5% Y^3+^ doping.

The optical limiting and two photon absorption coefficients of pure CdMoO_4_ and Y doped CdMoO_4_ nanostructures were investigated *via* the open aperture (OA) z scan technique using a pulsed Nd: YAG laser having a wavelength of 532 nm with a repetition rate of 10 Hz. Samples (pure and doped) with equal weights were dispersed in the same volume of diethylene glycol. The OA z-scan experiments, conducted with the samples in a quartz cuvette (path length = 1 mm), showed 70% transmittance for all of the samples. As shown in Fig. 7, the open aperture (OA) z-scan transmittance intensity decreases as the sample moves towards the focus and then the transmittance increases as it moves away from the focus, depicting a signature of reverse saturable absorption (RSA), where the excited state absorption cross section is greater than the ground state absorption cross section. Experimental data were fitted theoretically (eqn (1)) to understand the involved nonlinear optical absorption process. Here, the experimental data were found to fit well with the equation of two photon absorption given by1
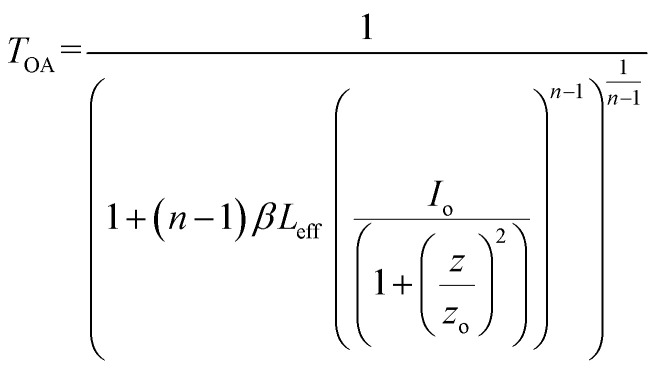
where *T*_OA_ corresponds to transmittance in open aperture mode, *β* is the effective nonlinear absorption, and *L*_eff_ and *I*_o_ are the effective path length and input irradiance, respectively.^41–43^ The increase in the depth of the valley pattern (Fig. 7) with Y doping concentration confirms the influence of yttrium in the enhancement of nonlinear optical (NLO) behaviour of CdMoO_4_. The attained 2PA coefficient (*β*) values of pure CdMoO_4_ and the Y doped CdMoO_4_ nanostructures are depicted in Table 1, from which it is evident that the 2PA efficient is enhanced upon yttrium doping. Here, doping of yttrium into cadmium molybdate results in the incorporation of Y^3+^ ions into the Cd^2+^ lattice and as the valence states of Y and Cd are different, as confirmed by the XPS spectral analysis of Fig. 5, this leads to the creation of new oxygen vacancies. Therefore, as the Y dopant concentration increases, the transfer of energy or electrons from the conduction band of the metal increases, which results in an enhanced 2PA coefficient.^44–46^ Similar work on the optical limiting properties and PA of Au coated CdS nanoparticles was reported by Mathew *et al.* and it was concluded that coupling of the local field effect by surface plasmon resonance (SPR) and excitonic oscillator strength of the CdS nanoparticles contributes to an enhancement in the optical nonlinearity.^47^ In our case, the enhancement in nonlinearity is due to the generation of impurity energy levels and creation of oxygen defects caused by substitution of Y^3+^ ions into the CdMoO_4_ matrix. The formation of oxygen vacancies can introduce dopant energy levels, which extract the photogenerated electrons from the conduction band, thereby increasing the photo-absorption and separation rate of charge carriers. On the other hand, the oxygen vacancy sites can act as electron-rich centres that increase the photo-absorption capacity of the material.

**Table 1:** The nonlinear optical parameters of the Y^3+^doped CdMoO_4_ nanostructures

Sample	Nonlinear two photon absorption coefficient, *β* (× 10^−11^ m W^−1^)	Onset of optical limiting threshold (× 10^12^ W m^−2^)	Optical limiting threshold (× 10^13^ W m^−2^)
Pure CdMoO_4_	2.4	8.88	3.9
0.1% Y^3+^–CdMoO_4_	2.9	8.47	3.0
0.3% Y^3+^–CdMoO_4_	3.5	7.62	2.5
0.5% Y^3+^–CdMoO_4_	8.9	2.98	2.3

An intensity dependent open aperture z-scan was performed for the pure CdMoO_4_ nanostructures and 0.5% Y^3+^ doped CdMoO_4_ (ESI Fig. S1 and 2†). The obtained two-photon absorption coefficients for the pure CdMoO_4_ nanostructures are 2.4 × 10^−11^ m W^−1^, 2.1 × 10^−11^ m W^−1^ and 1.8 × 10^−11^ m W^−1^ at an on-axis input intensity of 2.46 × 10^12^ W m^−2^, 1.23 × 10^12^ W m^−2^, and 0.61 × 10^12^ W m^−2^, respectively. For 0.5% Y^3+^ doped CdMoO_4_, the determined two-photon absorption coefficients at 2.46 × 10^12^ W m^−2^, 1.23 × 10^12^ W m^−2^, and 0.61 × 10^12^ W m^−2^ are 8.9 × 10^−11^ m W^−1^, 8.2 × 10^−11^ m W^−1^, and 7.1 × 10^−11^ m W^−1^, respectively. The two-photon absorption coefficients of the pure CdMoO_4_ nanostructures and 0.5% Y^3+^ doped CdMoO_4_ vary with on-axis input intensity, which can be ascribed to the sequential two photon absorption. Hence, these results confirm the involvement of excited state absorption in the pure and Y^3+^doped CdMoO_4_ nanostructures.

**Fig. 8:**
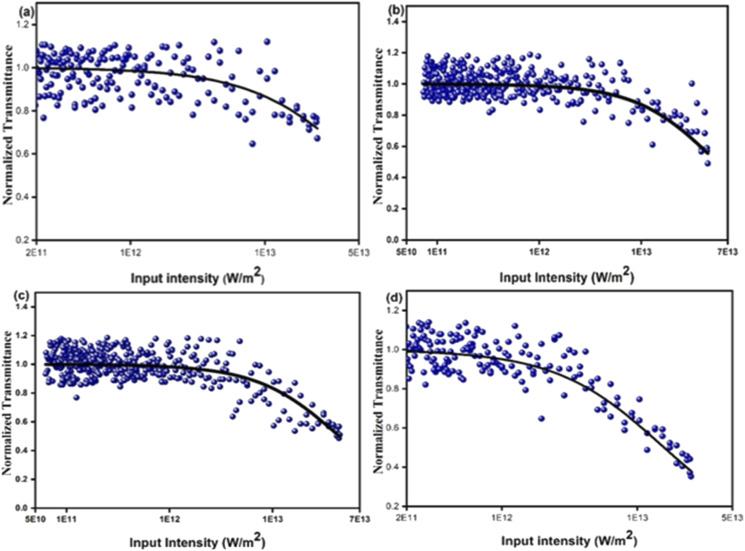
Optical limiting graph of Y^3+^ doped CdMoO_4_ nanostructures: (a) pure CdMoO_4_, (b) 0.1%, (c) 0.3% and (d) 0.5% Y^3+^ doping.

Optical power limiters are in high demand currently due to the increased usage of high-power lasers in different fields. Optical power limiting devices reduce transmittance at high power, whereas they completely transmit laser light at low input power or intensities. An ideal optical limiter should possess high linear transmittance, high stability, and a low limiting threshold.^48–51^Fig. 8 shows optical limiting traces for the pure and yttrium doped CdMoO_4_. The observed energy absorbing based optical limiting can be ascribed to two photon absorption and the estimated onset optical limiting threshold is given in Table 1. A good optical limiting response was observed for the higher concentrations of yttrium doped CdMoO_4_ nanoparticles compared to the pristine CdMoO_4_, which is due to the generation of impurity energy levels and creation of oxygen defects caused by an increase in Y^3+^ ion substitution in the CdMoO_4_ matrix resulting in a lower optical limiting threshold. The comparision of 2PA with its energy for different laser exicitation with Y-doped CdMoO_4_ nanostructures is depicted in Table 2.^52^

**Table 2:** Literature comparison of two-photon absorption coefficient with its energy with different laser excitations

Sample	Laser	NLO response	*β* cm W^−1^	Ref.
MoS_2_ (ref. 48)	532 nm, 5 ns	RSA	0.75 × 10^−8^	48
CdFe_2_O_4_ (ref. 53)	532 nm, 50 mW	2 PA	5.87 × 10^−3^	53
C–N–S-doped TiO_2_ NPs (ref. 54)	532 nm, 9 ns, 10 Hz	2 PA + ESA	2.12 × 10^−8^	54
Cu_2_O (ref. 55)	532 nm, 5 ns	2 PA + ESA	6.4 × 10^−9^	55
CdMoO4 (present work)	532 nm, 5 ns	2 PA	2.4 × 10^−8^	

## Conclusion

We have successfully synthesised pure and yttrium doped (at different concentrations: 0.1, 0.3 and 0.5%) cadmium molybdate nanostructures using a co-precipitation method. The XRD patterns confirmed the formation of CdMoO_4_ with a tetragonal structure of scheelite type and that incorporation of dopants through substitution does not alter the crystal structure but decreased the grain size. This was further confirmed by Raman spectroscopy from which 13 Raman active modes were identified and all other modes matched well with the literature. Both pure CdMoO_4_ and Y doped CdMoO_4_ possess a cubic morphology and lattice contraction resulted in a reduction in particle size. UV-Vis absorption showed that the addition of Y^3+^ enhances light absorption in the visible region and the band gap increases upon an increase in dopant concentration. The nonlinear properties investigated using the z-scan technique using a 532 nm pulsed Nd: YAG laser showed that the pure CdMoO_4_ and Y^3+^ doped CdMoO_4_ nanostructures exhibit two photon absorption induced optical limiting and that the nonlinear absorption coefficient increases with yttrium concentration owing to the increase in oxygen defects. Compared to the pure CdMoO_4_ nanostructures, Y^3+^ doped CdMoO_4_ can be used as a potential candidate for optical limiting applications in the development of laser safety devices.

## Conflicts of interest

There are no conflicts to declare.

## Supplementary Material

RA-012-D2RA04687C-s001
