# Supplementary material for: Influence of yttrium doping on the nonlinear optical limiting properties of cadmium molybdate nanostructures
Source: RSC Adv. 2022 Sep 23;12(42):27145–53. doi: 10.1039/d2ra04687c (PMC9503379; doi:10.1039/d2ra04687c)
Supplement: RA-012-D2RA04687C-s001 [file RA-012-D2RA04687C-s001.pdf]

Electronic Supplementary Material (ESI) for RSC Advances.
This journal is © The Royal Society of Chemistry 2022

Supplementary data

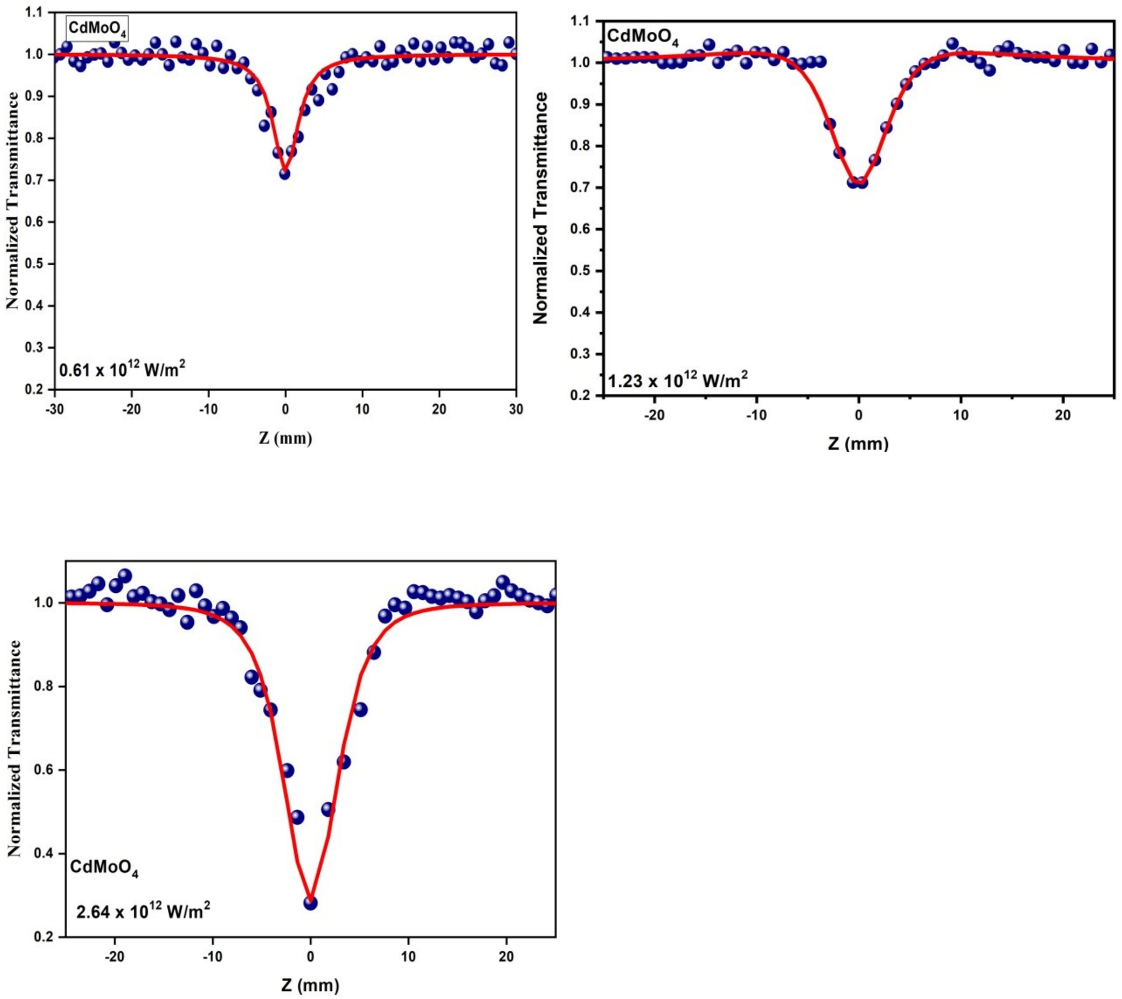

Fig. 1 Intensity dependent open aperture Z-scan graph for pure $CdMoO_4$ nanostructures

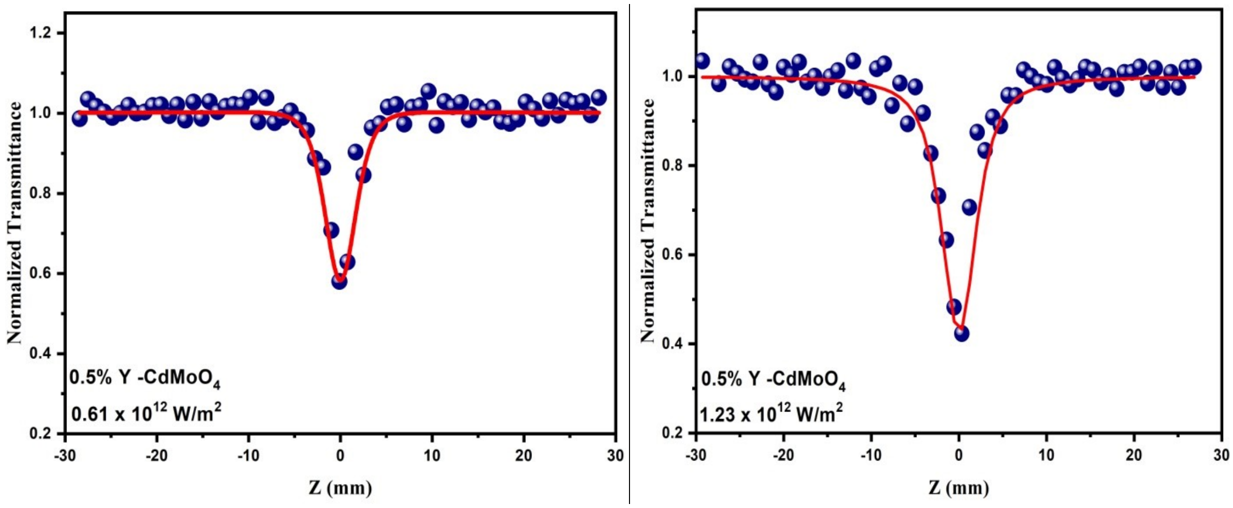

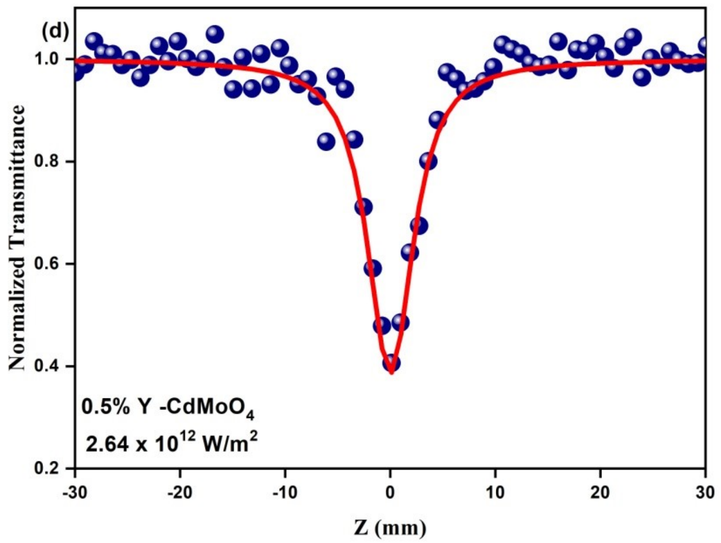

Fig. 2 Intensity dependent open aperture Z-scan graph for 0.5 % Y^{3+} doped $CdMoO_4$ nanostructures
